# Physiotherapeutic Rehabilitation for a Geriatric Patient With Discitis Associated With Pott’s Spine: A Case Report

**DOI:** 10.7759/cureus.57083

**Published:** 2024-03-27

**Authors:** Nandini V Zore, H. V. Sharath, Nikita Gangwani

**Affiliations:** 1 Department of Paediatric Physiotherapy, Ravi Nair Physiotherapy College, Datta Meghe Institute of Higher Education and Research (DU) Sawangi Meghe, Wardha, IND; 2 Department of Musculoskeletal Physiotherapy, Ravi Nair Physiotherapy College, Datta Meghe Institute of Higher Education and Research (DU) Sawangi Meghe, Wardha, IND

**Keywords:** spine rehabilitation, infective discitis, vertebral discitis, pott’s spine with neurodeficit, back pain

## Abstract

Discitis linked to Pott’s spine is an infrequent yet severe issue, especially difficult to manage among elderly individuals due to age-related bodily changes and concurrent health issues. This report details the successful physiotherapy-based recovery of a senior patient afflicted with discitis related to Pott’s spine. The individual, a 61-year-old man, presented symptoms including intense back pain, restricted movement, and neurological issues. The diagnosis was confirmed via imaging scans, indicating spinal tuberculosis and vertebral disc involvement. Treatment embraced a comprehensive approach involving medication alongside physiotherapy. The physiotherapeutic regimen aimed at pain alleviation, enhancing spinal flexibility, strengthening weakened muscles, and promoting functional autonomy. Techniques such as manual therapy, targeted exercises, and patient education were employed. Despite the challenges posed by the patient’s age and existing conditions, significant enhancements in pain management, mobility, and everyday functioning were noted during the rehabilitation journey. This case underscores the significance of prompt diagnosis, collaborative care, and personalized physiotherapeutic interventions in attaining positive outcomes for elderly patients grappling with discitis associated with Pott’s spine. Further investigation is needed to delineate optimal rehabilitation approaches for this intricate condition among the elderly.

## Introduction

This case report delves into the physiotherapeutic rehabilitation of a geriatric patient diagnosed with discitis linked to Pott’s spine. Discitis, although rare, poses significant challenges in treatment, especially among the elderly population, due to age-related physiological changes and concurrent health issues. Pott’s spine, characterized by vertebral tuberculosis, further complicates the condition, often necessitating a multidisciplinary approach for effective management. In this context, physiotherapy emerges as a crucial component, focusing on pain management, restoration of mobility, strengthening weakened musculature, and enhancing functional independence. The successful rehabilitation of this 61-year-old male patient highlights the importance of tailored physiotherapeutic interventions in addressing the complexities of discitis associated with Pott’s spine in geriatric individuals.

Discitis is an infection of the intervertebral disc space that can lead to paralysis, sepsis, epidural abscess, or other life-threatening complications if left untreated and may sometimes present with limited laboratory abnormalities to clue in a diagnosis [1]. Discitis is a condition characterized by infection within the intervertebral disc. Multisystem evaluation of these patients is crucial since de novo spinal infections are uncommon and most typically result from the hematogenous spread of infection from other parts of the body [2]. The aim of this study is to report the successful physiotherapeutic rehabilitation of a geriatric patient diagnosed with discitis associated with Pott’s spine.

Nonoperative treatment with antibiotics is typically the approach for managing discitis. However, in cases where surgery is necessary, debridement and intervertebral fusion are commonly performed to facilitate healing, minimize neurological damage, and restore spinal stability [3]. The majority of patients affected by discitis often have underlying predisposing conditions, such as alcoholism, diabetes mellitus, HIV infection, spinal abnormalities, potential systemic or local sources of infection, or a combination of these factors. Patients exhibiting neurological symptoms like muscle weakness, altered sensation in the lower extremities, and bladder or bowel dysfunction should undergo a thorough neurological assessment, including tests for sensation, muscle strength (using the 5-point MRC scale), intrinsic muscle reflexes, and nerve-stretching assessments [4,5]. Spinal infections caused by bacteria can manifest as either pyogenic or granulomatous, including diseases such as tuberculosis or brucellosis. These infections can impact any part of the spinal column, resulting in various diagnostic terms such as spondylodiscitis or discitis. Furthermore, the infection can extend within the spinal canal, involving the dural sac or the epidural space, or spread to the soft tissues surrounding the vertebrae [6-8].

The multidisciplinary collaboration among healthcare professionals, including physiotherapists, plays a pivotal role in optimizing patient care and facilitating rehabilitation progress [9,10]. Moreover, it emphasizes the need for further research to explore optimal rehabilitation approaches tailored to the unique needs of elderly patients with discitis associated with Pott’s spine. By elucidating the successful rehabilitation journey of this geriatric patient, this report contributes to enhancing understanding and guiding future interventions in managing similar cases effectively.

## Case presentation

Patient information

A 61-year-old male farmer was brought to Acharya Vinoba Bhave Rural Hospital (AVBRH). He was apparently alright four months ago when he developed pain in his lower back region, which was acute in onset, dull aching, moderate in intensity, gradually progressive, radiating to bilateral hip and knee joints, aggravated while walking, and relieved on rest and medications. Now, he has difficulty doing his daily activities. The patient is operated on for micro-depression at L3-L4. He was on antiretroviral therapy (AKT) but stopped three times. He had undergone certain investigations, like an MRI, X-ray, and CT scan, and was diagnosed with infective discitis at C5-C6, L3-L4, and D11. H/O of weight loss and fever are present. Presently, the patient complains of low backache with weakness in the lower limb. On December 30, 2023, the patient underwent spinal fixation at D10-L1 levels and decompression at D11-12 levels.

Clinical finding

Before commencing the examination, the patient provided informed consent, following which he underwent assessment. The individual, aged 61, exhibited an ectomorphic physique. He demonstrated compliance with instructions, alertness, cooperative behavior, and a clear orientation to time and place. Additionally, the patient showed no fever and maintained stable hemodynamics. During observation, the patient was found resting in a supine position with knee and ankle support provided by cushions, though occasionally observed lying on one side. The patient reported experiencing weakness in the lower limbs. A neurological examination revealed intact sensory responses but diminished muscle tone and strength in both lower extremities. Furthermore, all deep tendon reflexes were attenuated, and a Babinski sign was elicited. The patient's functional abilities were found to be entirely reliant on assistance.

Motor examination

In conducting a motor examination for a geriatric patient with discitis secondary to Pott's spine, we focus on assessing the patient's range of motion (Tables 1-2).

**Table 1:** Pre-treatment range of motion AROM: active range of motion; ROM: range of motion

Sr No.	ROM	Pre-treatment AROM	
		Right (degrees)	Left (degrees)
1	Cervical		
	Flexion	40	45
	Extension	50	70
	Right lateral rotation	45	45
	Left lateral rotation	45	45
2	Shoulder		
	Flexion	0–170	0–170
	Extension	0–60	0–50
	Abduction	0–165	0–160
	Internal rotation	0–65	0–70
	External rotation	0–75	0–70
3	Elbow		
	Flexion	0–145	0–145
	Extension	0	0
4	Wrist		
	Flexion	0–75	0–75
	Extension	0–65	0–65
5	Lumbar		
	Lateral flexion	0–15	0–15
	Rotation	0–3	0–3
6	Hip		
	Flexion	0–130	0–130
	Extension	0–20	0–20
	Abduction	0–25	0–30
	Adduction	25–0	30–0
	Internal rotation	0–30	0–35
	External rotation	0–25	0–30
7	Knee		
	Flexion	0–120	0–125
	Extension	120–0	125–0

**Table 2:** Post-treatment range of motion ROM: range of motion; PROM: passive range of motion

Sr No.	ROM	Post-treatment PROM	
		Right (degrees)	Left (degrees)
1	Cervical		
	Flexion	40	45
	Extension	50	70
	Right lateral rotation	45	45
	Left lateral rotation	45	45
2	Shoulder		
	Flexion	0–170	0–170
	Extension	0–60	0–50
	Abduction	0–165	0–160
	Internal rotation	0–65	0–70
	External rotation	0–75	0–70
3	Elbow		
	Flexion	0–145	0–145
	Extension	0	0
4	Wrist		
	Flexion	0–75	0–75
	Extension	0–65	0–65
5	Lumbar		
	Lateral flexion	0–15	0–15
	Rotation	0–3	0–3
6	Hip		
	Flexion	0–170	0–175
	Extension	0–45	0–45
	Abduction	0–40	0–40
	Adduction	40–0	40–0
	Internal rotation	0–45	0–45
	External rotation	0–40	0–40
7	Knee		
	Flexion	0–135	0–135
	Extension	0	0

Muscle strength examination involves assessing the strength of key muscle groups in the upper and lower extremities using manual muscle testing (MMT) or other objective measures. Strength deficits may be present due to pain, disuse, or neurological involvement, as mentioned in Tables 3-4.

**Table 3:** Manual muscle testing Grade 0: no contraction observed or palpable; Grade 1: trace contraction felt or observed, but no movement occurs; Grade 2: movement occurs, but not against gravity; Grade 3: movement occurs against gravity but not against resistance; Grade 4: movement occurs against some resistance, but not against maximal resistance; Grade 5: normal strength; movement occurs against maximal resistance.

Sr No.	MMT	Upper limb (pre-treatment)	
		Right	Left
1.	Shoulder		
	Flexors	3/5	3/5
	Extensors	3/5	3/5
	Abductors	3/5	3/5
	Adductors	3/5	3/5
	Internal rotators	3/5	3/5
	External rotators	3/5	3/5
2.	Elbow		
	Flexors	3/5	3/5
3.	Wrist		
	Flexors	3/5	3/5
	Extensors	3/5	3/5
4.	Hip		
	Flexors	3/5	3/5
	Extensors	3/5	3/5
	Abductors	3/5	3/5
	Adductors	3/5	3/5
5.	Knee		
	Flexors	3/5	3/5
	Extensors	3/5	3/5
4.	Ankle		
	Dorsiflexion	2/5	3/5
	Plantarflexion	2/5	3/5

**Table 4:** Post-manual muscle testing Grade 0: no contraction observed or palpable; Grade 1: trace contraction felt or observed, but no movement occurs; Grade 2: movement occurs, but not against gravity; Grade 3: movement occurs against gravity but not against resistance; Grade 4: movement occurs against some resistance, but not against maximal resistance; Grade 5: normal strength; movement occurs against maximal resistance.

Sr No.	MMT	Upper limb (pre-treatment)	
		Right	Left
1.	Shoulder		
	Flexors	4/5	4/5
	Extensors	4/5	4/5
	Abductors	4/5	4/5
	Adductors	4/5	4/5
	Internal rotators	4/5	4/5
	External rotators	4/5	4/5
2.	Elbow		
	Flexors	4/5	4/5
3.	Wrist		
	Flexors	4/5	4/5
	Extensors	4/5	4/5
4.	Hip		
	Flexors	4/5	4/5
	Extensors	4/5	4/5
	Abductors	4/5	4/5
	Adductors	4/5	4/5
5.	Knee		
	Flexors	5/5	5/5
	Extensors	5/5	5/5
4.	Ankle		
	Dorsiflexion	5/5	5/5
	Plantarflexion	5/5	5/5

Investigation

**Figure 1:**
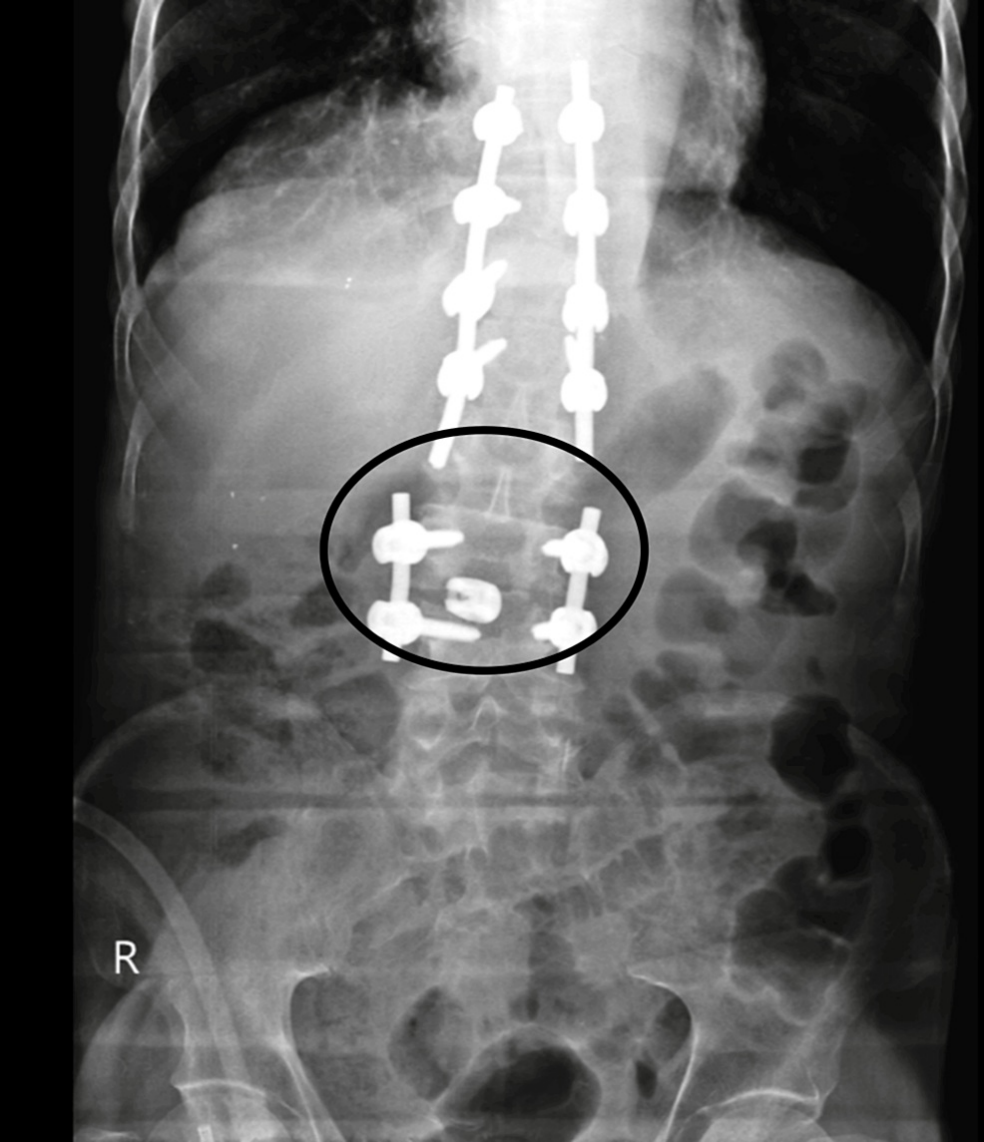
Micro-depression at L3-L4

An MRI of the lumbar spine reveals that the height and signal intensity of the vertebral bodies and intervertebral discs are normal. On the level of the disc, bulges diffuse moderately, comprising the right and mildly compromising the left neural canal. Collapse of the D11 vertebra. Lytic lesions are seen in the T12, L3, and L4 vertebrae. The visualized skeleton shows degenerative changes. Features suggest symptoms of Pott’s spine in Figure 1.

Physiotherapeutic intervention

Through comprehensive evaluation, including observation of posture and gait, range of motion (ROM) assessment, strength testing, neurological examination, and functional assessment, the aim is to identify impairments and limitations that may impact mobility and function. This information guides the development of a tailored intervention plan (Table 5), which includes therapeutic exercises, manual therapy techniques, pain management strategies, assistive devices, and patient education, with the overarching goal of improving mobility, reducing pain, restoring function, and enhancing the patient's overall quality of life.

**Table 5:** Rehabilitation AFO'S: ankle foot orthosis; ACBT: active cycle breathing techniques

	Goals	Intervention	Intensity
				Phase 1: (day 1 to 1 week)	Psychosocial support and education	Emotional support was provided across diverse settings, complemented by peer support	Regular sessions based on patient needs.
To reduce pain	Electrotherapy modalities like IFT (interferential therapy)	Duration-15 min					
			Unable to perform bed mobility					Functional mobility activities like rolling and weight shifting	(Day 1 to 1 week)
										To stretch the muscle	Intercoastal muscle stretching	10 repetitions with 3 set
Manual stretching: a physiotherapist may perform manual stretching techniques by applying gentle pressure or traction to the ribcage, helping to lengthen and stretch the intercostal muscles.	10 repetitions with 3 set											
		Rib mobilization: physiotherapists may use hands-on techniques to mobilize the ribs, promoting better movement and flexibility of the intercostal muscles.	10 repetitions with 3 set									
					Foam rolling the sides of the torso can aid in releasing tension and tightness in the intercostal muscles, fostering relaxation and flexibility.	10 repetitions with 3 set				
							Stretching exercises: specific stretching exercises targeting the intercostal muscles, such as side stretches or thoracic stretches, can help improve flexibility and reduce muscle tightness.	10 repetitions with 3 set		
							Phase 2: (1 weeks to 2 weeks)	To strengthening upper limb muscles	Isometric strengthening exercises progressive to active assisted exercises for core muscle strengthening, strengthening of core muscles external oblique muscles, rectus abdominis, transversus abdominis muscles; to perform during first 2 weeks.		10 repetitions with 3 set
												Phase 3: (2 to 3 weeks)	Gait training	Proprioceptive training and gait training with a walker, including navigating obstacles, are integrated into the rehabilitation program.	1 round In day
																Mobility and transfers	Top of form	As much as possible per session
																			Phase 4: (4 to 6 weeks)	Improving balance and coordination	For improving static balance, lateral weight shifting, spot marching, and maintaining balance.	10 repetitions with 3 sets
		Pre-ambulatory training	Physical therapy support is provided as necessary to assist with knee stabilization while walking between parallel bars with bilateral AFOs.																				For 10 minutes in day (1 to 2 weeks)
Maintaining good ventilation	Deep breathing exercises, thoracic expansion exercises, and active cycle breathing technique.															10 repetitions with 3 set							
					Phase 5: (after 6 weeks)	Coordination Frenkel exercises											Sit to stand exercises were given in standing position: walking forward Walking backwards Walking in zig-zag walking sideways and returning to the original position.	10 repetitions with 3 sets					

Outcome measures 

In evaluating the effectiveness of physiotherapeutic intervention for a geriatric patient with discitis secondary to Pott's spine, outcome measures (Table 6) encompassing pain intensity, functional mobility, quality of life, and physical function are paramount. These measures may include functional independence measures (FIM), the geriatric depression scale (GDS), and Tinnetti, and they all provide insights into individual functional limitations and goals. Regular monitoring of these outcome measures enables clinicians to gauge the patient's progress, tailor interventions accordingly, and ultimately optimize functional outcomes and overall well-being.

**Table 6:** Outcome measures FIM: functional independence measures; GDS: geriatric depression scale; LEFS: lower extremity functional scale

Sr.	Scale	Pre-treatment	Post-treatment
1.	Tinetti scale	5/28	21/28
2.	FIM	2	6
3.	GDS	11/15	5/15
4	LEFS	30/80	70/80

## Discussion

In the context of physiotherapeutic rehabilitation for geriatric patients with discitis associated with Pott’s spine, multidisciplinary collaboration and individualized treatment plans are paramount. This case underscores the importance of a comprehensive approach that addresses not only the specific musculoskeletal challenges posed by discitis but also considers the broader health concerns common in geriatric populations. By focusing on pain management, mobility improvement, muscle strengthening, and functional independence enhancement, physiotherapy plays a central role in the rehabilitation process. Moreover, the successful outcome observed in this case highlights the effectiveness of tailored physiotherapeutic interventions in mitigating the adverse effects of discitis and Pott’s spine in elderly patients, thereby improving their overall quality of life and functional status [11,12].

Determining the etiological agent of infectious discitis, a rather prevalent cause of morbidity, is a problem. The hallmark symptom of discitis is persistent back pain, typically worsening at night and progressing from mild discomfort to severe, excruciating pain, especially after initial post-surgical pain relief. This pain may radiate to various areas, such as the buttocks, thighs, legs, scrotum, groin, or perineum [13-16]. Additional symptoms like fever, fatigue, and malaise may also occur at varying frequencies (11-68% of cases). Despite successful early surgical intervention in controlling infection and providing rapid pain relief, patients may still encounter difficulties, particularly if significant vertebral damage leads to spinal instability and kyphotic deformity. However, conservative treatment generally yields favorable long-term outcomes, with success rates ranging from 70% to 83%.

The hematogenous spread of spinal infection in this patient is most likely facilitated through an artery or venous blood vessel. Distinguishing between degenerative disc disease, characterized by endplate destruction, and spinal infection, characterized by disc space constriction, osteophytosis, and reactive sclerosis of the endplates, is relatively straightforward. Conventional CT scans have limited utility in diagnosing early spondylitis and disc space infections. Magnetic resonance imaging (MRI) is the preferred imaging modality for detecting spinal infections, enabling targeted treatment with organism-specific antibiotics and spinal immobilization, often resulting in positive long-term outcomes [17,18]. Postoperative back pain in spine patients is commonly attributed to postoperative vertebral osteomyelitis and post-procedural discitis, particularly affecting elderly and immunocompromised individuals. In cases of discitis, MRI typically reveals decreased intensity on T1-weighted imaging and increased or equivalent intensity on T2-weighted imaging two to six weeks post-surgery, with bone marrow edema frequently observed in the L4 region. Core stability exercises may significantly benefit individuals with nonspecific chronic low back pain by improving quality of life, activating and strengthening core muscles, and reducing pain intensity and functional impairment [19,20].

Furthermore, the discussion emphasizes the need for ongoing research to refine and optimize rehabilitation strategies for similar cases. Future studies should explore the efficacy of various physiotherapeutic modalities, such as manual therapy, therapeutic exercises, and patient education, in improving outcomes for geriatric patients with discitis associated with Pott’s spine. Additionally, investigating the long-term impact of physiotherapy on pain management, mobility, and functional capacity in this population could provide valuable insights into the sustainability of rehabilitation interventions. By continuously refining our understanding and approaches to physiotherapeutic rehabilitation in geriatric patients with complex spinal conditions, healthcare professionals can better address the unique needs and challenges of this population, ultimately improving patient outcomes and quality of life.

## Conclusions

In conclusion, this case report highlights the successful physiotherapeutic rehabilitation of a geriatric patient diagnosed with discitis associated with Pott’s spine. Through a multidisciplinary approach incorporating pharmacological management and tailored physiotherapeutic interventions, significant improvements were observed in pain levels, mobility, and functional capacity. The positive outcome underscores the importance of early diagnosis, collaborative care, and personalized rehabilitation strategies in achieving favorable outcomes for elderly patients with complex spinal conditions. Moving forward, further research is warranted to explore optimal rehabilitation approaches and long-term outcomes in this population. By continuing to refine our understanding and approaches to physiotherapeutic rehabilitation, healthcare professionals can better address the unique needs of geriatric patients with discitis associated with Pott’s spine, ultimately enhancing their quality of life and functional independence.
